# Diversity and inclusion in clinical trials: Evolution throughout the development of an mRNA COVID-19 vaccine

**DOI:** 10.3389/fpubh.2023.1113003

**Published:** 2023-04-26

**Authors:** Jameka Hill, Diane Montross, Melanie Ivarsson

**Affiliations:** Moderna, Inc., Cambridge, MA, United States

**Keywords:** coronavirus – COVID-19, SARS-CoV-2, equity, clinical trial, representation, mRNA-1273 vaccine

## Abstract

Despite the importance of equitable representation in clinical trials, disparities persist with racial and ethnic minorities remaining largely underrepresented in trial populations. During the coronavirus disease 2019 (COVID-19) pandemic, wherein disease disproportionately affected racial and ethnic minority groups, the necessity for diverse and inclusive representation in clinical trials has been further highlighted. Considering the urgent need for a safe and efficacious vaccine, COVID-19 vaccine clinical trials faced marked challenges in rapidly enrolling participants without forgoing diverse representation. In this perspective, we summarize Moderna’s approach toward achieving equitable representation in mRNA-1273 COVID-19 vaccine clinical trials, including the COVID-19 efficacy (COVE) study, a large, randomized, controlled, phase 3 trial of mRNA-1273 safety and efficacy in adults. We describe the dynamics of enrollment diversity throughout the COVE trial and the need for continuous, efficient monitoring and rapid pivoting from initial approaches to address early challenges. Insights gained from our varied and evolved initiatives provide key learnings toward achieving equitable representation in clinical trials, including establishing and listening to a Diversity and Inclusion Advisory Committee, repeatedly engaging with key stakeholders on the necessity for diverse representation, creating and disseminating inclusive materials to all trial participants, establishing methods to raise awareness for interested participants, and enhancing transparency with trial participants to build trust. This work shows that diversity and inclusion in clinical trials can be attained even in the most extreme circumstances and highlights the importance of efforts toward building trust and empowering racial and ethnic minorities with the knowledge to make informed medical treatment decisions.

## Introduction

Equitable representation in clinical trials remains essential for the public health sector, particularly for countries with racially pluralistic societies (1). An understanding of how age, race, gender, underlying medical conditions, and other factors can affect an individual’s response to a particular medication, vaccine, or medical treatment is critical for guiding effective patient healthcare (1, 2). Although regulatory efforts have been undertaken to promote clinical study populations that are reflective of population demographics (3–6), racial and ethnic minorities remain largely underrepresented (7, 8). Historically underrepresented groups, including those of Hispanic or Latino, Black or African American, or Asian descent, comprise approximately 40% of the population in the United States (9), but the vast majority (76%) of the 292,766 participants in US clinical trials between 2015 and 2019 were White (10). These gaps in representation may prevent generalization of trial findings to the entire spectrum of the intended patient populations and could preclude scientific advances to certain at-risk individuals who could particularly benefit from the trial outcomes. Moreover, a long history of racial inequity and documented human rights violations has led to a general distrust of healthcare institutions and medical research among racial and ethnic minorities (11, 12), further hampering their representation in clinical research.

The importance of health equity and ensuring that diverse patient populations are included in clinical trials was further emphasized during the coronavirus disease 2019 (COVID-19) pandemic (13, 14), caused by infection with severe acute respiratory syndrome coronavirus 2 (SARS-CoV-2). At the start of the pandemic, multiple reports from the United States indicated that older adults and certain ethnic and racial minorities were disproportionately affected by SARS-CoV-2 (15–19). In particular, the burden of COVID-19 fell primarily on US communities disparately affected by structural and social inequities, including Black or African Americans, Native Hawaiians and Pacific Islanders, American Indians and Alaska Natives, and Hispanic or Latinx individuals (15, 16, 19–22). Case and mortality rates for COVID-19 among these populations were higher than might be expected based on demographic representation (15, 21, 22); for example, despite Black Americans representing 31% of patients in a Louisiana-based health system, a retrospective cohort study reported that 77% of its COVID-19 hospitalized patients and 71% of its patients who died in the hospital were Black (18). Notably, the disproportionate burden of COVID-19 among these groups is not reflective of a particular genetic predilection to severe disease, but instead stems from longstanding healthcare inequities and disparate social determinates of health among these populations (23).

Vaccination against SARS-CoV-2 remains one of the best options toward controlling the ongoing COVID-19 pandemic, including those populations disparately affected by the disease. To this effect, equitable representation in clinical trials that evaluate the safety and efficacy of COVID-19 vaccines dictates that enrollment should include those populations at highest risk of infection or those disproportionately burdened by disease. Herein we summarize the multifaceted and evolving approaches undertaken by Moderna, Inc., to ensure diversity and inclusion in the study populations of phase 3 trials of mRNA-1273 (SPIKEVAX; Moderna, Inc., Cambridge, MA) (24), an mRNA-based vaccine against SARS-CoV-2 that received Emergency Use Authorization (EUA) from the US Food and Drug Administration (FDA) for use in individuals ≥6 months of age, as well as full approval in individuals ≥18 years of age as of January 2022 (25). This report focuses on the dynamics of enrollment diversity from the pivotal phase 3 COVID-19 efficacy (COVE) trial conducted in US adults (NCT04470427), which enrolled participants aged 18 years and older whose locations or circumstances put them at appreciable risk of exposure to SARS-CoV-2 infection and COVID-19 (26, 27). We describe our strategies toward achieving a study population representative of US demographics, which faced additional hurdles amid the need to enroll participants at a historic pace without compromising equitable representation. Further, we highlight the need for efficient monitoring of trial population dynamics that helped guide a major change from initial approaches to ensure historically underrepresented populations were adequately represented in the final trial population. Ultimately, we describe how these recruitment strategies were similarly applied to phase 3 clinical trials on mRNA-1273 safety and efficacy in healthy US adolescents aged 12 to 17 years (TeenCOVE; NCT04649151) (28) and healthy US children aged 6 months to 12 years (KidCOVE; NCT04796896) (29).

## Initial recruitment approaches for the COVE study

The initial enrollment strategy for the COVE study was designed with input from Moderna, Inc., Operation Warp Speed leadership, and the COVID-19 Prevention Network (CoVPN), the latter of which was formed by the National Institute of Allergy and Infectious Diseases (NIAID) to develop and conduct COVID-19 vaccine and monoclonal antibody phase 3 studies. Further, in accordance with guidance from the FDA (30), the COVE study aimed to enroll populations at high risk of SARS-CoV-2 infection and at risk for severe COVID-19. As such, the original trial protocol stated that between 25% and 40% of enrolled participants would be either aged ≥65 years, or <65 years and at risk for severe COVID-19 illness (the specific conditions considered high risk are described in the published protocol (26)). Overall, the clinical trial was designed to enroll participants aged 18 years and older whose locations or circumstances put them at appreciable risk of infection and severe disease and aimed to have appropriate representation across factors of age, sex, race, risk of SARS-CoV-2 exposure (i.e., occupational risk), and risk of severe COVID-19 illness (based on protocol-specified predefined underlying conditions).

Participants were enrolled from a broad geographic distribution of 99 research study sites in 31 states across the United States, with site selection based on the site’s feasibility to conduct the study, locally high incidence of COVID-19 based on epidemiologic data and predictive modeling, and site proximity to places of high occupational risk (e.g., factory and construction workers or grocers). Study sites were also selected based on each site’s queried ability to enroll diverse and at-risk populations, and discussions with sites indicated their overall awareness and understanding of the importance of this task.

**Figure 1:**
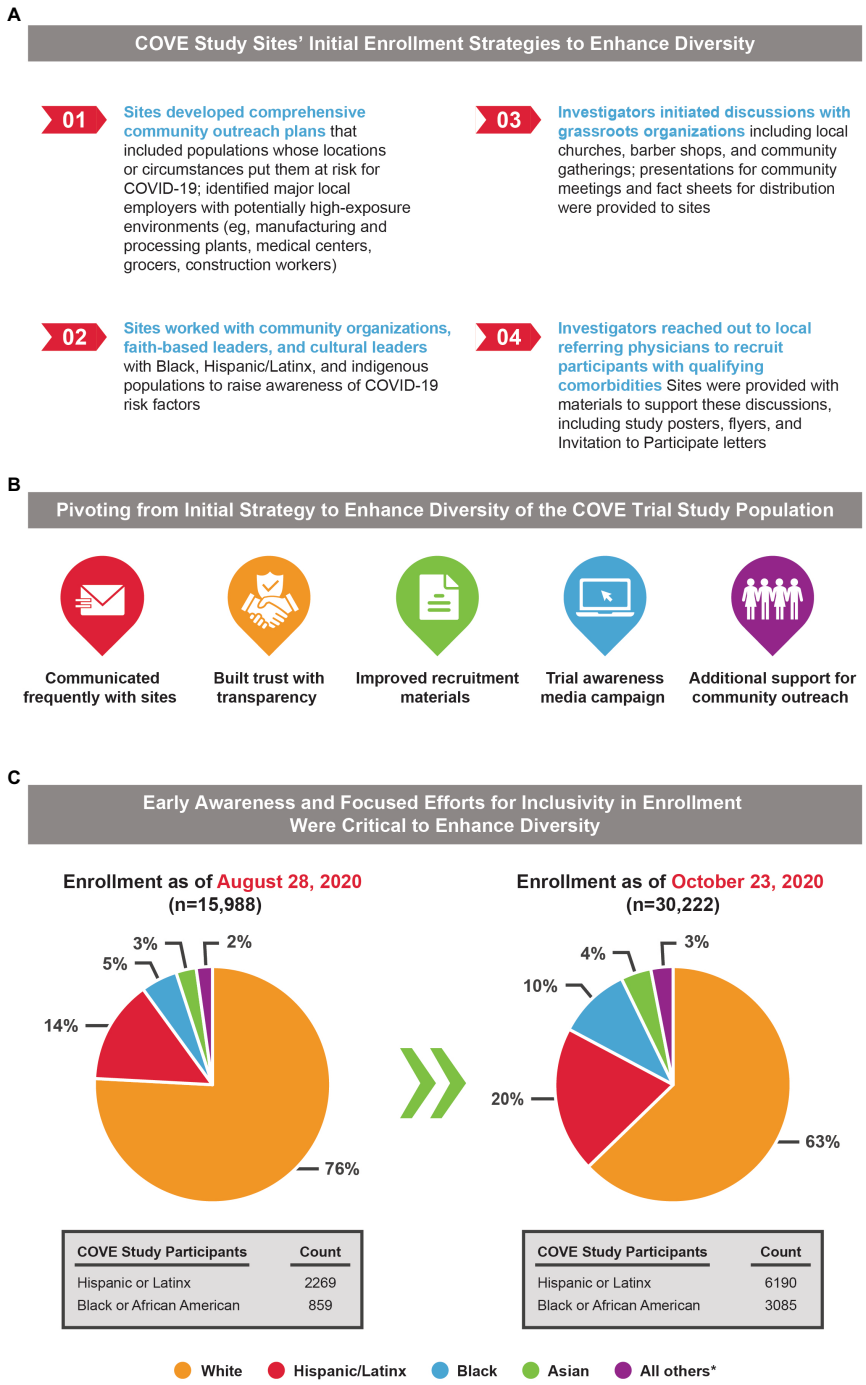
Initial enrollment strategy of the COVE study and the need for an evolving recruitment approach to achieve equitable representation in study populations. **(A)** The initial enrollment strategy for the COVE study was designed to target an inclusive and diverse study population reflective of the intended patient population. **(B)** Diverse representation initially lagged in the COVE study, and a multifaceted approach to address this unexpected challenge was implemented to help the study achieve equitable representation. **(C)** Pie charts present percentages of COVE participants within each demographic characteristic and highlight the under-representation of Black and Hispanic/Latinx Americans during the earlier timepoints of the trial, which was addressed by a pivoting from initial strategies. *Includes American Indian or Alaska Native, Native Hawaiian or other Pacific Islander, multiracial, not reported and unknown, or other.

The initial enrollment strategy also took into consideration the longstanding barriers to enrollment of Black and Hispanic participants in US clinical trials, with industry and community experts brought in to review the strategy and to monitor enrollment progress. Initially, sites were asked to develop a local enrollment strategy that supported and planned community outreach tactics for their unique location (Figure 1A), which included: (1) developing comprehensive community outreach plans for populations whose locations or social circumstances placed them at risk for COVID-19, (2) working with Black and Hispanic community organizations, faith-based leaders, and cultural leaders to raise risk awareness, (3) study investigators initiating discussions with grassroots organizations, and (4) study investigators reaching out to local referring physicians and community health partners to recruit participants with qualifying comorbidities, using supportive materials such as informational trial brochures, flyers, and “Invitation to Participate” letters.

## An initial lag in racial and ethnic group enrollment and subsequent revision of the recruitment strategy to ensure equitable representation

The first participant was enrolled into the COVE study on July 27, 2020, at 6:00 am and enrollment proceeded thereafter at an unprecedented pace. However, as enrollment progressed, it became clear that while representation across diverse groups was achieved, a substantially lower percentage of Black, Hispanic, and American Indian or Alaska Native trial participants were enrolled than anticipated based on US population demographics.

Discussions with study sites were initiated to understand the potential reasons for low daily enrollment of racial and ethnic minorities despite initial site selection data indicating enrollment of these groups was feasible. COVE investigators indicated to Moderna that the social and political unrest in the United States during the summer of 2020 augmented deep-rooted fear and distrust of the government, the pharmaceutical industry, and clinical trial sites within historically underrepresented populations. These issues ultimately manifested as increased hesitation to enroll into the COVE study, highlighting health disparities and underpinning the need to address health equity within the drug development process.

To tackle these specific enrollment hurdles facing racial and ethnic minorities in the COVE study, a Diversity and Inclusion Advisory Committee comprised of members from the National Institutes of Health, Operation Warp Speed, faith-based leaders, the CoVPN, and the US Department of Veterans Affairs was established. The committee was tasked to review race and ethnicity enrollment demographics on a weekly basis, review current community outreach activities and outcomes, develop strategies to ensure all communities significantly impacted by COVID-19 were participating, and support development and implementation of participant retention strategies (Figure 1B). In addition, the clinical study protocol was amended on August 20, 2020, in response to feedback from the FDA Center for Biologics Evaluation and Research to explicitly state that an aim was to enroll a representative sample of racial and ethnic minorities.

Enrollment data were also utilized to rapidly revise the recruitment strategy to ensure equitable representation among the COVE study population. Enrollment metrics were monitored daily, with transparent and regular updates shared with study sites regarding current enrollment objectives and metrics, in addition to changes in the enrollment strategy. Weekly calls that highlighted data from enrollment dashboards also aided recruitment efforts, with site personnel commenting that these dashboards provided a highly useful level of data granularity not previously seen. Dashboards were also key to raising awareness since they included a snapshot of how each site’s enrollment data compared to local census data. This approach cultivated shared accountability among the sites.

In addition, meetings were held with public health authorities and Moderna to speak with the study sites on multiple occasions to provide encouragement and reinforce the importance of achieving a dataset that was representative of the US population. Moderna provided sites with training on conscious inclusion to ensure a full understanding of barriers to minority enrollment and prepared sites for engagement in complex, culturally-sensitive conversations to address concerns among potential African American and Latinx trial participants.

Ultimately, delineating these expectations and establishing open communication with the COVE study sites helped to quickly change the recruitment strategy in order to address those initial challenges that hampered an appropriately diverse study population. This revised strategy encompassed multiple key aspects that together ensured equitable representation in the COVE trial. One tactic was to leverage clinical trial registries of individuals who previously provided self-identified race and ethnicity information and who had pre-consented to receive information regarding COVID-19 clinical trials. One such registry, CoVPN, was used by 60 of the 99 sites. In addition, Moderna, Inc., partnered with a network of patient-recruitment companies, trusted national pharmacy networks, healthcare organizations, and patient advocacy groups to recruit individuals from diverse backgrounds.

A third aspect of the revised strategy was to add 10 additional study sites in late August 2020, and Moderna, Inc., also encouraged the establishment of CoVPN satellite sites, such as the nonprofit Casa de Maryland in Baltimore that was headquartered in an epicenter of the COVID-19 pandemic and had a strong relationship with the local community. Further, site-facing materials were revamped for participant inclusivity from diverse populations, and new materials were created and translated into Spanish to support study staff’s continued focus on clinical trial diversity. Finally, study sites collaborated with local community leaders to disseminate additional information on a local level to dispel widespread rumors and misinformation regarding the mRNA-1273 investigational vaccine.

**Figure 2:**
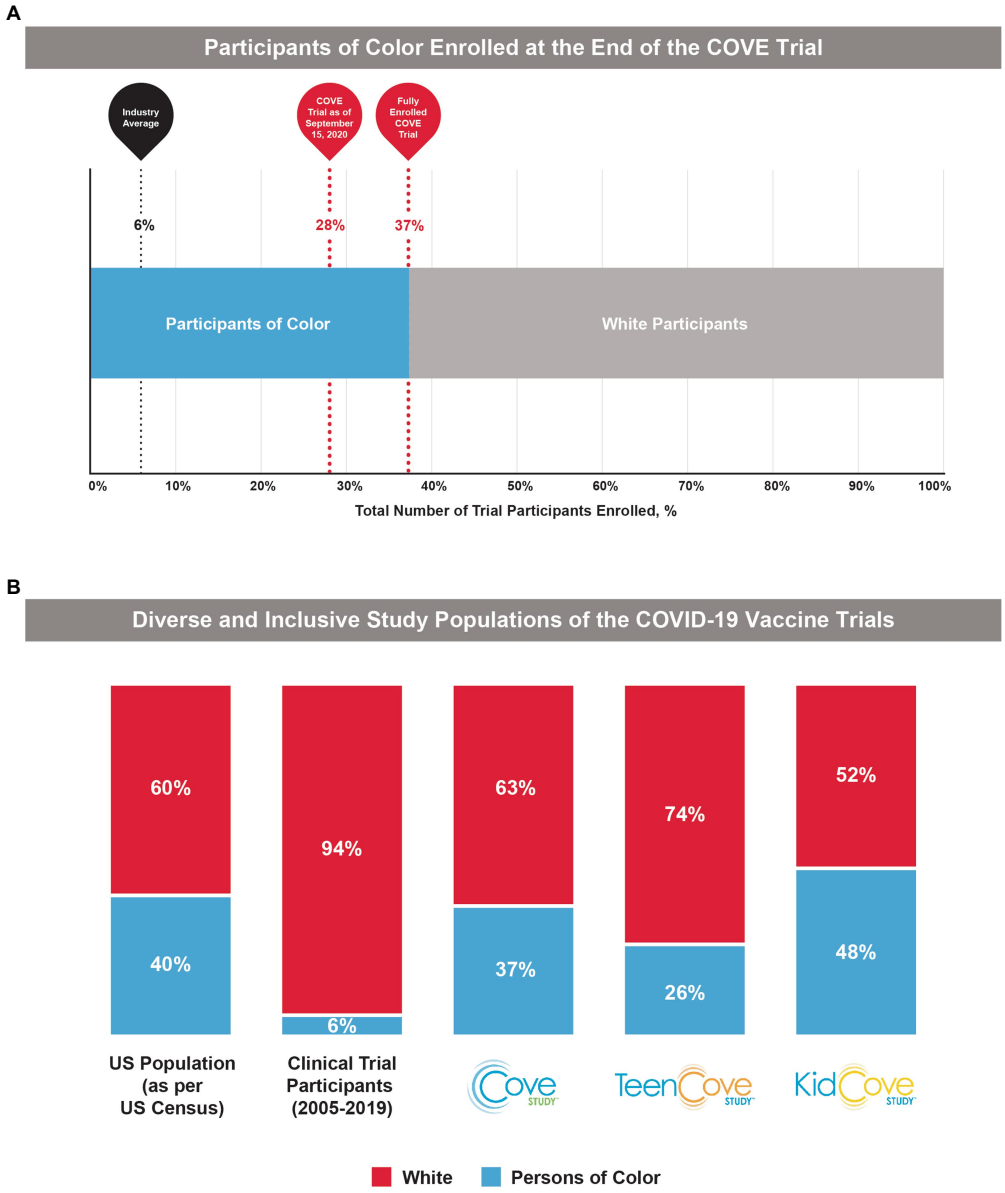
Participant demographics in the COVE, TeenCOVE, and KidCOVE studies. **(A)** The percentage of Participants of Color enrolled in the COVE trial increased after pivoting from initial strategies, but notably was above the industry standard (6%) even at earlier trial timepoints. **(B)** In comparison to previous clinical trial populations from 2005 to 2019 (10), the phase 3 mRNA-1273 studies (COVE, TeenCOVE, and KidCOVE) were representative of demographics for Persons of Color in the United States (9). Persons of color were defined as individuals who identify as, but not limited to, Native American, Alaska Native, Indigenous, Latinx, Hispanic, Asian, Pacific Islander, African American, Black, Middle Eastern, and two or more races.

To further ensure that the final COVE trial population reflected US demographics, Moderna made the necessary decision in early September 2020 to slow enrollment of White participants to allow for adequate enrollment of underrepresented individuals. Although this decision slowed the pace of enrollment, Moderna and the US government understood that achieving a diverse study population was essential to ensure a more robust and representative body of clinical knowledge and build confidence in the generalizability of the safety and efficacy data collected in the COVE study. This adjustment to the enrollment strategy proved invaluable in ensuring the final COVE study population was representative of US demographics. The combination of these strategies, along with the unwavering commitment of the investigators and operations teams to extend the enrollment opportunity to historically underrepresented populations, and provide the information needed to make an informed decision, led to increased representation of Black and Hispanic/Latinx participants (Figures 1C, 2A).

## Achieving equitable representation in the final study population

In October 2020 when enrollment in the COVE study completed, the final study population was representative of the entire spectrum of the intended treatment population in the United States (Figure 1C). As of the EUA data cut-off date (November 15, 2020), 47% of the participants in the full analysis set were female and 25% were aged ≥65 years. Overall, participants of color comprised 37% of the COVE study population, which was similar to current US population demographics (40%) and was drastically higher than historically achieved in prior clinical trials (6%; Figure 2B). A majority of the participants (82%) were considered at occupational risk for SARS-CoV-2 exposure, with 25% of participants being healthcare workers. Participants at high risk for severe COVID-19 comprised 22% of participants.

These recruitment strategies were similarly applied for the TeenCOVE and KidCOVE trials. There were upfront and concerted efforts with trial sites to enroll participants from historically underrepresented groups, and both trials set deliberate demographic objectives. The TeenCOVE trial was rapidly commenced after the COVE trial to include vulnerable population targets, ultimately enrolling 26% participants of color, with an even distribution of male (51%) and female (49%) participants (28). These enrollment strategies were then further applied to the KidCOVE trial, which ultimately enrolled 48% participants of color, with a similar distribution of male (51%) and female (49%) participants (29). Further, the KidCOVE trial enrolled a similar percentage of Hispanic participants as the COVE trial (19% and 21%, respectively). Overall, these results highlight the application of lessons learned during the COVE study toward recruitment strategies in following studies, which was accomplished with moderate success for TeenCOVE and further success for KidCOVE.

## Lessons learned for future clinical development

The challenges experienced during the execution of the COVE enrollment strategy yielded important lessons about effective measures to achieve equitable representation among study participants that helped inform enrollment strategies for TeenCOVE and KidCOVE. Specifically, despite the vetted protocol design, site selection, and clear plan to enroll participants across the intended treatment population, the initial enrollment strategy of the COVE trial fell short in enrolling Black and Hispanic individuals. The precise underlying reason is multifaceted and could include increased fear of exploitation within these communities, which may have been exacerbated by the social unrest of events in 2020. After Moderna, Inc., became aware of the insufficient enrollment of Black and Hispanic individuals, all efforts were made to mobilize a data-directed, multipronged strategy to address the observed racial and ethnic discrepancies, which included utilizing registries and strategic satellite sites as well as adding sites with established means to recruit underrepresented minorities.

It is essential that the data collected in clinical studies on COVID-19 vaccines such as mRNA-1273 can be reflective of entire populations, especially those at high risk of severe COVID-19 illness. The COVID-19 pandemic has disproportionately affected Black, Hispanic, and Native American populations who suffer from greater COVID-19–related morbidity and mortality than White Americans. Importantly, these disparities among racial and ethnic minorities do not appear to be due to a genetic predisposition to severe COVID-19 disease; rather, the same inequalities in the US healthcare system that have led to increased rates of underlying medical conditions, which also correlate with socioeconomic status, are shown to be risk factors for severe COVID-19 (18, 31).

Moving forward, the healthcare industry must continue to allocate resources to empower and build trust among diverse communities at a grassroots level. Mistrust as well as lack of knowledge or awareness of clinical trials and their importance have been previously identified as critical barriers to diverse clinical trial representation (32). Leaders in historically underrepresented groups have shared that gaining this trust will require recruiting and training more physicians and nurses (or allied healthcare professionals) with diverse backgrounds. The pharmaceutical industry must also work toward building an equitable partnership with diverse communities, while equitably developing safe and effective drugs and vaccines. The FDA published guidance in 2020 to ensure sponsors are considering diversity of study populations throughout clinical trial conduct (33). The data collected from all subgroups during the clinical trial phase should allow a more thorough assessment of subgroup-specific safety and efficacy signals before the medical product is administered post-approval. Most recently, the FDA also issued recommendations for industry in April 2022 to improve diversity in clinical trials by submitting a diversity action plan for phase 3 or pivotal trials (34).

In the midst of developing a COVID-19 vaccine in an unprecedented time, Moderna rapidly addressed initial enrollment challenges to refocus their strategy and ensure equitable representation in the final study population. As a result of these initiatives, healthcare professionals and patients can better understand the safety and efficacy of the vaccine in diverse populations. Moreover, vaccine efficacy data by demographic population are also available (26), which can aid in interrogating the benefits and risks of the vaccine. Beyond enrollment, it is also important to note that following the FDA issuance of the EUAs for COVID-19 vaccines, participants in the COVE, TeenCOVE, and KidCOVE studies were notified of their vaccine assignment and placebo recipients were eligible to instead receive mRNA-1273. These actions provide credence to how a sponsor can take an ethical route during a global pandemic setting and work toward addressing the historical mistreatment of medical atrocities for underrepresented groups. Moderna is also implementing these lessons learned from the COVE trial to multiple global vaccine development programs, including those for cytomegalovirus and respiratory syncytial virus, resulting in an average 36% of clinical trial participants being participants of color. However, inclusive representation in clinical trials is only a first step towards achieving health equity, with additional hurdles to ensuring equal vaccine access and receipt also remaining in the real world. Beyond clinical trials, further policy tactics will be needed for dismantling systemic racism within the healthcare network to allow for equitable opportunities for access to care and vaccination receipt (35); such strategies could include a comprehensive audit of diversity, equity, inclusion, and belonging (35) as well as keeping the lines of communication open to build trust, increase collaboration, and create tools and resources for those disproportionately impacted by disease (36).

This level of commitment to moral and scientific integrity highlights the real possibilities for patients and public health if industry leaders advocate for equitable healthcare. The pharmaceutical industry is encouraged to hold itself accountable to enroll the entire spectrum of intended treatment population in clinical trials, to perform rigorous safety and efficacy analyses by patient demographics, and to share the data in plain language with the public. These are foundational steps toward engaging with communities to foster trust and empowering people with the knowledge to make informed treatment decisions in partnership with healthcare professionals.

## Data availability statement

The original contributions presented in the study are included in the article/supplementary material, further inquiries can be directed to the corresponding author.

## Author contributions

All authors listed have made a substantial, direct, and intellectual contribution to the work and approved it for publication.

## Funding

This work was funded by Moderna, Inc.

## Conflict of interest

All authors are employees of Moderna, Inc., and hold stock/stock options in the company.

The authors declare that this review article received funding from Moderna, Inc. The funder was involved in the collection, analysis, interpretation of data, and the writing of this article or the decision to submit it for publication.

## Publisher’s note

All claims expressed in this article are solely those of the authors and do not necessarily represent those of their affiliated organizations, or those of the publisher, the editors and the reviewers. Any product that may be evaluated in this article, or claim that may be made by its manufacturer, is not guaranteed or endorsed by the publisher.
